# Metabolomic and Bacterial Community Signatures of Weathering Time in Empty Puparia of *Aldrichina grahami* (Aldrich, 1930) (Diptera: Calliphoridae)

**DOI:** 10.3390/insects17070736

**Published:** 2026-07-17

**Authors:** Xiangyan Zhang, Jiayi Wang, Sile Chen, Hai Wu, Xiaojiang Huang, Hongxiang Mao, Haiwen Xie, Chengxin Ye, Ming Liu, Yihong Qu, Yanjie Shang

**Affiliations:** 1 Department of Forensic Science, School of Basic Medical Sciences, Central South University, Changsha 410013, China; zxy196@csu.edu.cn (X.Z.); wangjy@csu.edu.cn (J.W.); 18216177266@163.com (S.C.); wuhai@csu.edu.cn (H.W.); yecx000@163.com (C.Y.); lmliuming0720@163.com (M.L.); 2 Public Security Bureau of Shaoyang, Shaoyang 422000, China; 17773920566@163.com (X.H.); 18207390849@163.com (H.M.); 3 Public Security Bureau of Loudi, Loudi 417000, China; 15873812017@163.com

**Keywords:** forensic entomology, minimum postmortem interval estimation, empty puparia weathering, metabolomics, bacterial community

## Abstract

Accurate postmortem interval estimation is an important issue in forensic investigations. Entomological evidence is widely used to estimate the minimum postmortem interval (PMI_min_), primarily based on insect development and succession on decomposing remains. However, once adult emergence has occurred and only empty puparia remain, the applicability of conventional development-based methods becomes limited. Empty puparia, which can persist at crime scenes for extended periods, may provide time-related information based on their weathering after adult emergence. Nevertheless, for *Aldrichina grahami* (Aldrich, 1930) (Diptera: Calliphoridae), a forensically important necrophagous species, systematic models for empty puparia weathering remain limited. In this study, a 13-month indoor exposure experiment was conducted to characterize weathering-associated changes in empty puparia of *A. grahami* using metabolomic and bacterial community analyses. The results showed that lipid metabolites, particularly long-chain fatty acids such as stearic and palmitic acids, decreased consistently with weathering time. In contrast, bacterial communities showed variation over time but exhibited weaker associations with weathering time. In internal cross-validation, the metabolomics model showed lower prediction error than the bacterial community model, while decision-level integration yielded the lowest error within this dataset. These results suggest that empty puparia undergo measurable molecular changes during indoor weathering, which may assist in estimating the post-eclosion interval (PEI) and thereby provide supplementary information for PMI_min_ assessment in long-interval cases.

## Simple Summary

Empty puparia are the cases left after adult flies emerge from their pupal stage. In long-interval death investigations, larvae or pupae may no longer be present, but empty puparia can still remain at the scene. If the age of empty puparia after adult emergence can be estimated, this information can be added to the time needed for the fly to develop on the body, helping investigators estimate the minimum time since death. In this study, empty puparia of *Aldrichina grahami* (Aldrich, 1930) (Diptera: Calliphoridae) were kept indoors for 13 months. Their chemical profiles and bacterial communities were analyzed over time. Lipid-related chemical changes showed clearer time-related patterns than bacterial changes, suggesting that empty puparia may provide useful information in long-interval forensic cases.

## 1. Introduction

Forensic entomology plays an important role in estimating the minimum postmortem interval (PMI_min_), particularly in cases involving advanced decomposition, with necrophagous flies constituting one of the principal sources of entomological evidence [1]. Development-based estimates generally reflect the PMI_min_ rather than the full interval elapsed since death, as insect colonization may be delayed after death [2]. During decomposition, these flies complete their development and emerge as adults, leaving behind large numbers of empty puparia at the scene [3]. Owing to their rigid structure and resistance to environmental factors, empty puparia can persist for extended periods at crime scenes. The progressive changes associated with their weathering over time provide information on the post-eclosion interval (PEI), defined as the interval between adult emergence and evidence collection, and may therefore complement PMI_min_ estimation [4]. In forensic casework, estimation of the PEI may help approximate the timing of adult emergence when the collection time of empty puparia is known. When combined with species-specific developmental data, this information may contribute to PMI_min_ assessment.

In natural environments, organic matter undergoes gradual weathering under the combined influence of environmental factors such as temperature, humidity, and biological activity, leading to time-dependent changes in chemical composition and structure [5,6]. Similar processes also occur in biological remains. In forensic entomology, puparia are protective structures formed by the contraction and sclerotization of the cuticle of late instar larvae. They are primarily composed of chitin and associated proteins, which confer a relatively high degree of stability [7,8]. However, when empty puparia are exposed to natural conditions over prolonged periods, their structural features and chemical composition may still gradually change under environmental influences, including temperature, humidity, and light [9]. Previous studies have indicated that these changes are, to a certain extent, correlated with exposure duration, suggesting their potential utility for estimating the PEI in long-interval cases [10].

In studies involving empty puparia as complementary evidence for PMI inference, Bajerlein et al. incorporated empty puparia as one of several entomological indicators within a development-based approach, in which the PMI_min_ was inferred from the age of the most advanced insect stage using thermal summation methods, alongside additional estimates derived from other taxa [11]. However, as pointed out by Wydra et al., PMI_min_ estimation based on empty puparia is subject to substantial uncertainty due to the unknown PEI, particularly under long-term or variable environmental conditions, which complicates the determination of the appropriate starting point for retrospective estimation of insect development [12]. Therefore, empty-puparial weathering primarily provides an estimate of the PEI, which should be combined with insect developmental information when used to support PMI_min_ assessment. In recent years, chemical analytical techniques have provided new avenues to address this limitation. Among these, studies focusing on cuticular hydrocarbons (CHCs) have been extensively investigated. Zhu et al. demonstrated through field experiments that the total abundance of CHCs in empty puparia decreases significantly with increasing exposure time, and that differences in degradation patterns among individual hydrocarbons may serve as potential indicators for assessing puparia weathering [13]. Paula et al. further confirmed that chemical profiling based on GC-MS can not only estimate weathering time but also distinguish empty puparia from different generations [14]. In addition, Sharif et al. reported that local microenvironmental conditions significantly influence chemical weathering processes, and that integrating machine learning approaches improves prediction accuracy [6]. Furthermore, Qu et al. applied Fourier transform infrared (FTIR) in combination with machine learning to model empty puparia weathering time, demonstrating that spectral features reflect the kinetics of chemical degradation and support rapid estimation of long-term puparial weathering intervals [15]. However, these studies mainly focused on CHCs or spectral features, whereas broader metabolomic alterations and bacterial community changes during empty-puparia weathering remain insufficiently characterized.

As organic remains, empty puparia may undergo degradation processes involving physical weathering, chemical transformations, and bacterial community changes [6,16]. Previous studies have indicated links between bacterial succession and changes in the chemical characteristics of decomposing biological remains [17]. However, in the context of empty-puparia weathering, temporal changes in bacterial communities and their associations with metabolite profiles remain to be systematically investigated.

The blowfly *Aldrichina grahami* (Aldrich, 1930) (Diptera: Calliphoridae) is a forensically important necrophagous species widely used in forensic entomology [18]. Due to its cold tolerance, which may broaden its seasonal occurrence and forensic applicability under cooler or sheltered conditions, together with locally derived developmental data, *A. grahami* is particularly relevant for indoor long-interval cases in this region, where persistent empty puparia may remain after larval evidence is no longer available [19,20]. In forensic practice, certain indoor death cases often involve individuals living alone or experiencing loss of contact with others, and may remain undiscovered for extended periods [21]. By the time of discovery, the body has often undergone several months or even longer decomposition, frequently reaching advanced decomposition or skeletonization [22]. Under such conditions, early postmortem indicators and insect developmental data commonly used for PMI_min_ estimation are often no longer applicable, leaving empty puparia in the surrounding environment as one of the few remaining sources of entomological evidence [10]. Therefore, investigating the indoor weathering of *A. grahami* empty puparia provides a basis for estimating the PEI and for supporting PMI_min_ assessment in long-interval cases.

Based on the above, the present study used empty puparia of *A. grahami* and combined bacterial community analysis with untargeted metabolomics to characterize changes during long-term weathering. By comparing bacterial community composition and metabolite profiles across weathering time, their temporal associations were evaluated. Furthermore, candidate time-associated molecular markers were identified to estimate the PEI of empty puparia and to evaluate its potential contribution to PMI_min_ assessment in long-interval cases.

## 2. Materials and Methods

### 2.1. Adult Rearing and Colony Maintenance

A laboratory colony of *A. grahami* was established from wild individuals collected from decomposing pig carcasses in Changsha, Hunan Province, China (28°12′ N, 112°58′ E), and had been continuously maintained under laboratory conditions for more than two years and more than six generations before the experiment. Species identification was confirmed using a combination of morphological and molecular methods. Morphological identification was performed according to the diagnostic criteria described in Corpse-Feeding Flies in China, and the species identity was confirmed by Prof. Lushi Chen (Guizhou Police Officer Vocational College) [23]. Molecular identification was conducted by amplifying the mitochondrial cytochrome c oxidase subunit I (COI) region using primers 658F (5′-GGTCAACAAATCATAAAGATATTGG-3′) and 658R (5′-RAAACTTCAGGRTGACCAAAGAATCA-3′). The resulting sequences were compared with reference sequences in the NCBI GenBank database using BLAST (https://blast.ncbi.nlm.nih.gov/Blast.cgi, accessed on 13 July 2026) to confirm species identity. The rearing chamber was maintained at 25.0 °C, 75% relative humidity, and a 12:12 h (light:dark) photoperiod. Adult flies were housed in nylon cages (15.0 × 15.0 × 10.0 cm) and provided with a mixture of milk powder and sucrose (1:1) in a 12 cm diameter dish. Water was supplied via cotton pads soaked in water and placed in containers within the cages. Fresh pig lung (15 g) was placed in containers inside the cages to stimulate oviposition. Egg masses laid within 2 h were collected and transferred to containers containing pig lung for incubation. When locomotor activity had markedly decreased and was nearly absent at the onset of pupariation, approximately 200 larvae were transferred together to a plastic pupation container (approximately 20.0 × 30.0 × 10.0 cm) containing a 2.0 cm layer of sterile sand. The pupation container was covered with breathable gauze and was not sealed. Once pupariation was complete, the pupae were removed from the sand and placed on nylon mesh until adult emergence.

### 2.2. Weathering Simulation and Sampling Procedure

Empty puparia were collected after adult emergence and used for long-term weathering experiments. Weathering time was defined as the interval after adult emergence. All empty puparia originated from a single emergence cohort and were exposed on an A4-sized nylon mesh frame (20.0 × 30.0 cm), without plastic containers or additional substrate. They were maintained under uncontrolled indoor conditions in a laboratory in Changsha, Hunan Province, China (28°12′ N, 112°58′ E). Outdoor temperature and relative humidity data recorded by a local meteorological station were included as reference indicators of regional seasonal trends (Figure S1). Under these conditions, the samples were protected from direct precipitation and solar radiation. Sampling was performed at 1, 3, 5, 7, 9, 11, and 13 months after adult emergence. This schedule was designed to cover indoor post-eclosion weathering from early to long-term intervals. At each time point, nine empty puparia were randomly selected and divided into three non-overlapping replicate pools, each consisting of three puparia. Each replicate pool was placed in a separate sterile 2 mL microcentrifuge tube. Bacterial community and metabolome analyses were performed on matched aliquots from the same replicate pool, ensuring one-to-one correspondence between the two omics datasets. All sampling procedures were conducted using sterile forceps. Immediately after collection, the samples were frozen in liquid nitrogen and stored at −80 °C for subsequent metabolomic and bacterial community analyses.

### 2.3. DNA Extraction, 16S rRNA Gene Sequencing, and Data Analysis

Genomic DNA was extracted from empty puparia samples using the E.Z.N.A. Soil DNA Kit (Omega Bio-tek, Norcross, GA, USA) according to the manufacturer’s instructions. The quality of the extracted DNA was assessed by 1% agarose gel electrophoresis, and DNA concentration and purity were measured using a NanoDrop 2000 spectrophotometer (Thermo Fisher Scientific, Wilmington, DE, USA). The extracted total DNA was used as the template for amplification of the V3-V4 hypervariable regions of the bacterial 16S rRNA gene. The primer pair 338F (5′-ACTCCTACGGGAGGCAGCAG-3′) and 806R (5′-GGACTACHVGGGTWTCTAAT-3′) was used for amplification. PCR reactions were performed in a 20 μL mixture containing 4 μL of 5× TransStart FastPfu Buffer, 2 μL of 2.5 mM dNTPs, 0.8 μL of each primer (5 μM), 0.4 μL of TransStart FastPfu DNA Polymerase, and approximately 10 ng of template DNA. The amplification program consisted of an initial denaturation at 95 °C for 3 min, followed by 27 cycles of denaturation at 95 °C for 30 s, annealing at 55 °C for 30 s, and extension at 72 °C for 30 s, with a final extension at 72 °C for 10 min. PCR products were verified by 2% agarose gel electrophoresis, and the target fragments were excised and purified. The purified products were quantified using a Qubit 4.0 fluorometer (Thermo Fisher Scientific, USA), followed by library construction using the NEXTFLEX Rapid DNA-Seq Kit (Bioo Scientific Corporation, Austin, TX, USA). The libraries were pooled in equimolar amounts and subjected to paired-end sequencing on an Illumina NextSeq 2000 platform (Illumina, San Diego, CA, USA).

Raw paired-end reads were demultiplexed and quality-filtered using fastp v0.19.6, and paired reads were merged using FLASH v1.2.11. Low-quality bases with quality scores below 20 were trimmed using a 50 bp sliding window, reads shorter than 50 bp after quality filtering and reads containing ambiguous bases were removed, and paired reads were merged when the minimum overlap length was 10 bp and the maximum mismatch ratio in the overlap region was 0.2. Samples were assigned according to barcode and primer sequences, allowing no barcode mismatches and a maximum of two primer mismatches. High-quality merged sequences were denoised with DADA2 in QIIME2 v2020.2, yielding 913,170 denoised sequences from 1,175,423 raw reads. After removing chloroplast and mitochondrial sequences, 727,822 reads remained, comprising 673 ASVs and 163 genus-level taxa; samples were then rarefied to 34,667 reads, with Good’s coverage ranging from 0.999885 to 1.000000. Taxonomic assignment was performed against the SILVA 16S rRNA gene database (v138) using a Naive Bayes classifier implemented in QIIME2. Alpha diversity indices, including Chao1 and Shannon indices, were calculated using mothur. Differences in alpha diversity among weathering intervals were first assessed using the Kruskal–Wallis test, followed by pairwise Wilcoxon rank-sum tests with Benjamini–Hochberg correction when appropriate. Principal coordinates analysis (PCoA) based on Bray–Curtis dissimilarity was performed to evaluate differences in bacterial community structure among samples, and permutational multivariate analysis of variance (PERMANOVA) was used to test group differences. Spearman correlation analysis was performed to identify bacterial taxa associated with weathering time. To account for multiple testing, *p* values were adjusted using the Benjamini–Hochberg false discovery rate (FDR) method. Bacterial genera with |r| > 0.6 and q < 0.05 were considered FDR-supported time-associated features. All analyses were conducted on the Majorbio Cloud Platform (https://cloud.majorbio.com, accessed on 10 May 2026). The sequencing project has been registered in the NCBI BioProject database under accession number PRJNA1464520.

### 2.4. GC-MS-Based Metabolic Profiling of Empty Puparia and Data Analysis

Untargeted metabolomic profiling was performed using a GC-MS-based workflow. For each pooled puparial sample, metabolites were extracted using a methanol–water/chloroform protocol. Briefly, the sample was transferred to a 2 mL grinding tube and mixed with 500 µL methanol–water solution (CH_3_OH:H_2_O, 4:1, *v*/*v*; methanol from Macklin, Shanghai, China) containing 0.02 mg/mL ribitol (P1069993, Damas-beta, Shanghai, China) as an internal standard. A steel ball and 200 µL chloroform (Macklin, Shanghai, China)were added, and the sample was frozen and ground at 50 Hz for 3 min twice. The extract was ultrasonicated at low temperature for 30 min, kept at −20 °C for 30 min, and centrifuged at 13,000 rcf and 4 °C for 15 min. The supernatant was transferred to a glass derivatization vial (ANPEL, Shanghai, China) and dried under nitrogen. Methoxyamination was performed by adding 80 µL methoxyamine hydrochloride in pyridine (15 mg/mL; Macklin, Shanghai, China), followed by vortexing for 2 min and incubation at 37 °C for 90 min. Subsequently, 80 µL BSTFA containing 1% TMCS (ANPEL, Shanghai, China) was added, followed by vortexing for 2 min and derivatization at 70 °C for 60 min. The derivatized samples were kept at room temperature for 30 min before GC-MS analysis.

GC-MS analysis was performed using a TRACE 1610 gas chromatograph coupled with an Orbitrap Exploris mass spectrometer (Thermo Fisher Scientific, USA) at Majorbio Bio-Pharm Technology Co., Ltd. (Shanghai, China). Derivatized samples were injected in split mode with an injection volume of 1 µL and a split ratio of 10:1. Separation was performed on a TG-5SILMS capillary column (30 m × 0.25 mm × 0.25 µm, 26096-1420, Thermo Fisher Scientific, USA), with high-purity helium as the carrier gas at a constant flow rate of 1.0 mL/min. The inlet temperature was 300 °C. The oven temperature was initially held at 80 °C for 0 min, increased to 310 °C at 20 °C/min, and then held for 8 min, with a total run time of 20 min. Mass spectrometry was performed using an electron impact ionization source at 70 eV. Data were acquired in full-scan mode over an *m*/*z* range of 35–500 with a mass resolution of 30,000 full width at half maximum. A pooled quality-control (QC) sample was prepared by mixing aliquots from all study samples and processed in the same manner as the analytical samples. Study samples were injected according to the weathering-time sequence rather than a randomized order. Three pooled QC injections were inserted across the analytical sequence, with QC1 before the study samples, QC2 between the Mon7 and Mon9 samples, and QC3 after the final study sample.

Raw GC-MS data were preprocessed using Compound Discoverer 3.3 SP3, including ion-peak filtering, deconvolution, peak matching, and feature extraction. Internal-standard peaks and known false-positive peaks, including noise, column-bleed peaks, and derivatization-reagent peaks, were removed from the data matrix, followed by redundancy removal and peak merging. Metabolites were putatively identified by matching mass spectra and retention indices (RI) against the NIST 2023 library, the Thermo Scientific GC-Orbitrap Metabolomics library, and an in-house Majorbio database. Alkane standards (C10–C33) were analyzed under the same chromatographic conditions for RI calculation. Candidate identifications were retained when the high-resolution filtering (HRF) score was >80, the match score was >600, and the absolute RI difference was <50. The resulting data matrix was uploaded to the Majorbio Cloud Platform (https://cloud.majorbio.com, accessed on 10 May 2026) for downstream analysis. Features with non-zero values in at least 80% of samples in at least one group were retained, missing values were imputed using the minimum value, and peak intensities were normalized by total-sum normalization. QC RSD values were calculated from pooled QC samples and reported for candidate metabolites. The normalized data matrix was log10 transformed before downstream analysis. PCA and PLS-DA were performed using the R package ropls (v1.6.2), and PLS-DA model stability was evaluated using cross-validation and a 200-permutation test. Differential metabolites were screened using VIP > 1 and *p* < 0.05. KEGG pathway annotation and Fisher’s exact test-based enrichment analysis were performed for annotated metabolites. In addition, Spearman correlation analysis was performed to identify metabolites associated with weathering time. To account for multiple testing, *p* values were adjusted using the Benjamini–Hochberg false discovery rate (FDR) method. Metabolites with |r| > 0.7 and q < 0.05 were considered FDR-supported time-associated features.

### 2.5. Bacterial Community–Metabolite Correlation Analysis

To evaluate the overall association between bacterial community structure and metabolite profiles during the weathering of empty puparia, Procrustes analysis was performed to assess the concordance between bacterial community and metabolome ordination results. Bacterial data were based on genus-level abundance matrices, while metabolomic data were derived from metabolite abundance matrices. PCA was performed separately on the genus-level and metabolite abundance matrices, and the first three components were used for Procrustes fitting. Procrustes analysis was performed using the vegan package (v2.4.3) in R (v3.3.1), and statistical significance was assessed by Monte Carlo permutation tests.

Based on the overall analysis at the feature level, key bacterial and metabolite features significantly associated with weathering time were selected. Procrustes analysis was then repeated using this subset of time-associated features to explore whether the two datasets showed similar time-related patterns.

In addition, partial Mantel analysis was performed to assess whether the correlation between the bacterial community and metabolome was independent of weathering time as a confounding factor. Distance matrices were constructed for both datasets, and the weathering time distance matrix was included as a controlling variable. Mantel correlation coefficients and their significance were assessed using permutation tests. Unless otherwise stated, *p* < 0.05 was considered statistically significant. All analyses were performed on the Majorbio Cloud Platform (https://cloud.majorbio.com, accessed on 10 May 2026).

### 2.6. Weathering-Time Prediction Analysis

Weathering-time prediction was performed using bacterial community, metabolome, and decision-level multi-omics datasets. Weathering time, corresponding to the experimentally controlled PEI after adult emergence, was treated as a continuous response variable corresponding to 1, 3, 5, 7, 9, 11, and 13 months. Only samples shared by the bacterial community and metabolome datasets were used for model construction, resulting in 21 pooled samples, with three pooled samples at each time point.

For the bacterial community model, genus-level count data were summed by genus, converted to relative abundance, and transformed as log10(CPM + 1). For the metabolome model, the preprocessed GC-MS metabolite abundance matrix was used, and quality-control samples were excluded before model fitting. Missing values were imputed using the median value calculated from the training set only.

Model performance was evaluated using leave-one-group-out cross-validation (LOGO-CV). In each outer fold, all three samples from one weathering time point were held out as the test set, while samples from the remaining six time points were used for training. Within each training set, non-constant features were ranked according to the absolute Spearman correlation between feature abundance and weathering time, and the top 15 features were retained for model fitting. Random forest regression models were constructed separately for the bacterial community and metabolome datasets using 800 trees, square-root feature sampling at each split, bootstrap sampling, and a fixed random seed.

For the multi-omics model, a decision-level late-integration strategy was used. Within each outer training set, nested leave-one-group-out predictions were first generated from the bacterial community and metabolome random forest models. These out-of-fold predictions were then used as two input variables to train a ridge regression meta-model. The final bacterial community and metabolome base models were refitted on the full outer training set and used to predict the held-out test samples, after which the ridge meta-model generated the integrated multi-omics prediction.

Prediction performance was summarized using MAE and R^2^ from pooled held-out test predictions, with 95% confidence intervals estimated by 10,000 weathering-time-point-level cluster-bootstrap resamples. Chance-level performance was assessed using 10,000 prediction-label permutations of the pooled held-out predictions. All machine-learning analyses were performed in Python (v3.14.5) using scikit-learn.

## 3. Results

### 3.1. Changes in Metabolite Abundance During Empty Puparia Weathering

GC-MS-based untargeted metabolomic analysis detected 154 metabolic features, of which 153 were putatively annotated. Among these, 83 had HMDB/LIPID MAPS annotations, 74 had KEGG compound IDs, and 52 of the KEGG-annotated metabolites were further mapped to KEGG pathways. These annotation categories overlapped and were not mutually exclusive.

**Figure 1:**
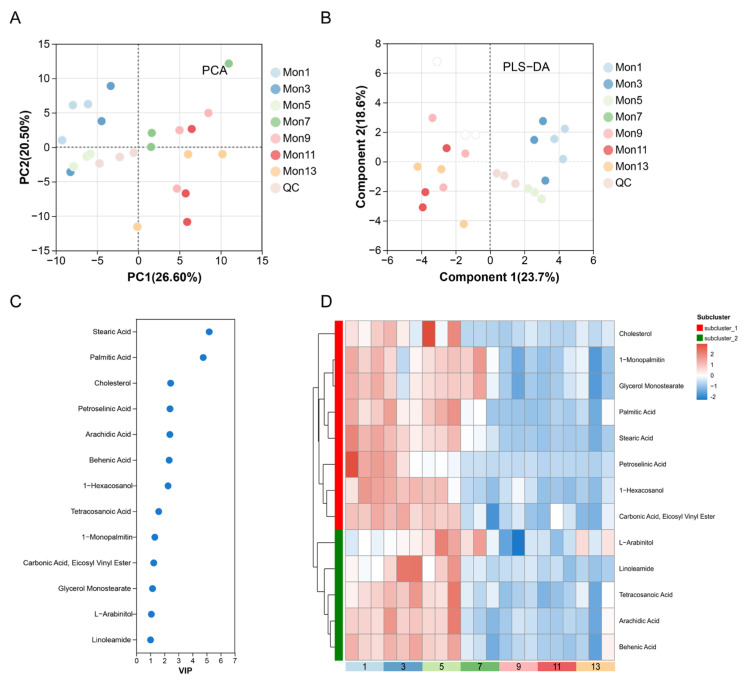
Metabolic profiling changes during empty puparia weathering. (**A**) Principal component analysis (PCA) score plot of metabolite profiles from puparial samples at different weathering intervals. (**B**) PLS-DA score plot of metabolite profiles across weathering time groups. (**C**) Variable importance in projection (VIP) scores of metabolites ranked in descending order, with a selection threshold of VIP > 1.0. (**D**) Heatmap of 13 candidate metabolites identified by the PLS-DA model. Hierarchical clustering was performed based on Z-score normalized relative abundances across different weathering intervals. The numbers above panel D indicate weathering intervals in months after adult emergence.

PCA was first used to provide an unsupervised overview of metabolomic variation. The first two principal components explained 26.60% and 20.50% of the total variance, respectively (Figure 1A). Early-stage samples tended to separate from mid- and late-stage samples, whereas overlap was observed among later time points. PLS-DA was then used as a supervised exploratory analysis. The first two PLS-DA components explained 23.70% and 18.60% of the X variance, respectively (Figure 1B). The fitted PLS-DA model yielded R^2^X(cum) = 0.423, R^2^Y(cum) = 0.217, and Q^2^(cum) = 0.0274 (Figure S2).

Based on the PLS-DA model and univariate testing, 13 candidate differential metabolites were identified using thresholds of VIP > 1.0 and *p* < 0.05 (Figure 1C). Among these, stearic acid, palmitic acid, and cholesterol showed the highest VIP scores. Hierarchical clustering based on Z-score-normalized relative abundances revealed that these metabolites, mainly long-chain fatty acids and their derivatives, exhibited an overall decreasing trend with increasing weathering time (Figure 1D). The full list of the 13 candidate differential metabolites, together with their identification information and direction of change, is provided in Supplementary Table S1. Raw peak intensity trends of the 13 candidate metabolites, including palmitic acid and stearic acid, are provided in Supplementary Table S2.

After Benjamini–Hochberg correction across all tested metabolites, 11 of the 13 candidate metabolites remained significantly associated with weathering time (|r| > 0.7 and q < 0.05; Supplementary Table S3). These time-associated metabolites were dominated by lipids and lipid-like molecules, including stearic acid, palmitic acid, octadecanamide, linoleamide, tetracosanol, and glycerol monostearate.

Further visualization of these 11 core metabolites revealed clear time-dependent patterns in their relative abundances during empty puparia weathering (Figure S3).

### 3.2. Bacterial Community Composition and Diversity During Empty Puparia Weathering

**Figure 2:**
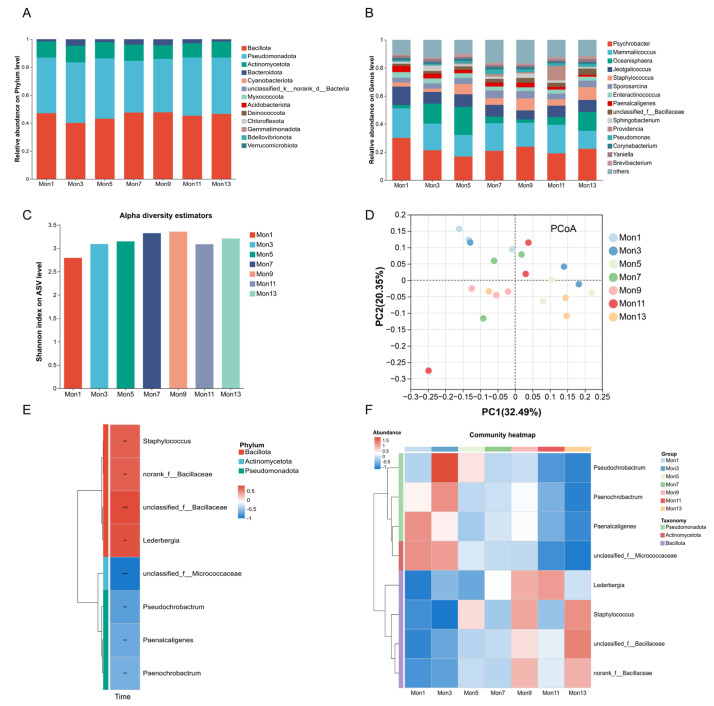
Bacterial community composition and succession during puparial weathering. Mon1, Mon3, Mon5, Mon7, Mon9, Mon11, and Mon13 indicate 1, 3, 5, 7, 9, 11, and 13 months after adult emergence, respectively. Bars in (**A**,**B**) show mean relative abundance across three pooled samples at each time point. (**A**) Relative abundance at the phylum level. (**B**) Relative abundance at the genus level. (**C**) Shannon diversity index. (**D**) Principal coordinates analysis (PCoA) based on Bray–Curtis distances. (**E**) Heatmap of bacterial genera showing nominal associations with weathering time. Asterisks indicate statistical significance: ** 0.001 < *p* ≤ 0.01, and *** *p* ≤ 0.001. (**F**) Spearman correlation coefficients of bacterial genera associated with weathering time.

At the phylum level, the bacterial community associated with empty puparia remained relatively stable throughout the entire weathering period (Mon1–Mon13), with stage-dependent fluctuations. Bacillota (Firmicutes) and Pseudomonadota (Proteobacteria) were consistently the dominant phyla, jointly accounting for more than 80% of the total relative abundance. The relative abundance of Bacillota slightly decreased at Mon3, followed by a gradual increase and stabilization from Mon7 to Mon13. The relative abundance of Pseudomonadota remained high, with only minor fluctuations. Actinomycetota (Actinobacteria) represented a stable third dominant phylum, whereas Bacteroidota (Bacteroidetes), Cyanobacteriota (Cyanobacteria), and other low-abundance phyla exhibited limited variation. Overall, no obvious replacement among phyla was observed, but rather dynamic shifts in the relative proportions of dominant taxa (Figure 2A).

At the genus level, more pronounced successional patterns were observed. Psychrobacter, Mammaliicoccus, and Jeotgalicoccus dominated the early stage (Mon1–Mon3), after which their abundances gradually decreased or fluctuated. Oceanisphaera showed the highest relative abundance at Mon5, suggesting a transient competitive advantage. Staphylococcus and Paenalcaligenes maintained low to moderate relative abundance, whereas Providencia and Pseudomonas showed increased relative abundance in the mid to late stages. The “others” category accounted for a substantial proportion across all time points, indicating the collective contribution of low-abundance taxa to community assembly (Figure 2B).

Overall, the bacterial community structure remained relatively stable, whereas dominant taxa exhibited stage-specific successional patterns. Early communities were dominated by environmentally tolerant taxa, while mid to late stages showed a shift toward putatively decomposition-associated taxa, reflecting dynamic ecological adaptation during long-term weathering.

The Shannon index varied across weathering time points but did not differ significantly among groups (Kruskal–Wallis test, *p* = 0.077, Figure 2C).

PCoA further revealed temporal separation of bacterial communities. Samples from Mon1-Mon3 clustered closely, indicating relatively stable early-stage community structure. With increasing weathering time, mid-stage samples became more dispersed, and late-stage samples were clearly separated from early-stage samples, suggesting compositional shifts in the bacterial community. PERMANOVA analysis showed significant differences among time groups (R^2^ = 0.2741, *p* = 0.007), indicating that bacterial community composition differed among weathering intervals under the tested indoor condition (Figure 2D).

Spearman correlation analysis identified several bacterial genera showing nominal associations with weathering time (Figure 2E,F). After Benjamini–Hochberg FDR correction, only unclassified_f_Micrococcaceae remained significantly associated with weathering time (r = −0.861, q = 0.016). Other genera, including unclassified_f_Bacillaceae, Lederbergia, norank_f_Bacillaceae, Staphylococcus, Pseudochrobactrum, Paenalcaligenes, and Paenochrobactrum, met the nominal correlation threshold but did not remain significant after FDR correction. Therefore, these taxa were interpreted as exploratory time-associated bacterial trends rather than robust FDR-supported markers. The correlation coefficients, *p* values, and q values for these genera are provided in Supplementary Table S4.

### 3.3. Bacterial Community–Metabolite Associations

**Figure 3:**
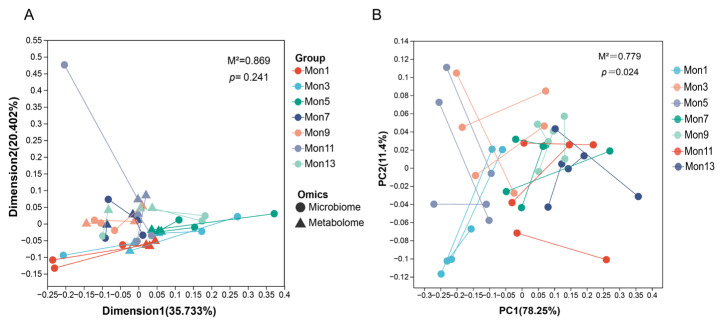
Procrustes analysis of bacterial community and metabolite profiles. Circles and triangles represent bacterial and metabolite ordinations from the same samples, respectively; connecting lines link paired positions of the same sample in the two ordinations. Shorter lines indicate greater concordance. M^2^ represents the Procrustes residual sum of squares, and *p* values were obtained by permutation tests. (**A**) Analysis based on all detected bacterial and metabolite features. (**B**) Analysis based on time-associated bacterial and metabolite features.

**Figure 4:**
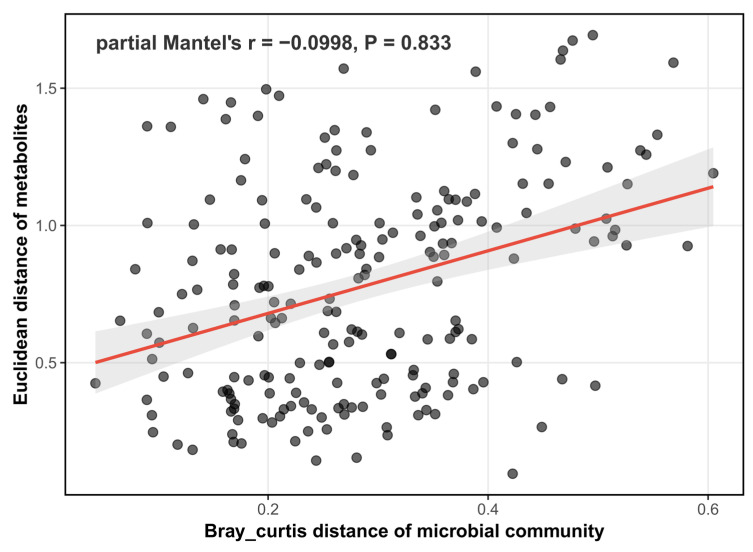
Partial Mantel analysis between bacterial community and metabolite profiles. Points represent pairwise bacterial and metabolite distances, the fitted line shows their association after controlling for weathering time, and the shaded area indicates the 95% confidence interval. r is the partial Mantel correlation coefficient, and *p* is the permutation-based significance level.

Procrustes analysis based on all detected bacterial and metabolite features showed weak and non-significant concordance between the two datasets (M^2^ = 0.869, *p* = 0.241; Figure 3A). After both datasets were restricted to time-associated features, the Procrustes result became significant (M^2^ = 0.779, *p* = 0.024; Figure 3B). However, because these features were selected based on the same variable, namely weathering time, this result was interpreted as shared time-related variation rather than evidence of a direct bacterial community–metabolite association. Consistently, partial Mantel analysis showed no significant association between the bacterial community and metabolite profiles after controlling for weathering time (r = −0.0998, *p* = 0.833; Figure 4). These results indicate that the two omics layers mainly showed parallel responses to weathering time or environmental exposure.

### 3.4. Weathering-Time Prediction Using Single-Omics and Decision-Level Multi-Omics Models

**Figure 5:**
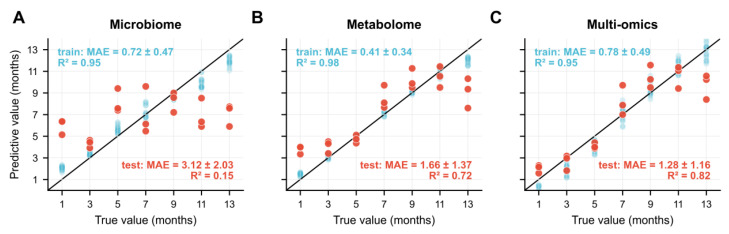
Prediction of empty puparia weathering time using single-omics and multi-omics machine-learning models. (**A**) Bacterial community-based random forest model. (**B**) GC-MS metabolomics-based random forest model. (**C**) Decision-level multi-omics model integrating bacterial community and metabolome predictions using ridge regression. Red points indicate held-out test predictions from leave-one-group-out cross-validation, and cyan points indicate training predictions from the corresponding outer folds. The black line represents the ideal 1:1 relationship between observed and predicted weathering time. MAE and R^2^ values are shown for training and held-out test predictions in each panel.

Machine-learning models showed clear differences in their ability to estimate empty puparia weathering time from omics profiles under the tested indoor condition. The bacterial community-based random forest model showed limited predictive performance, with a held-out test MAE of 3.12 months (95% CI, 1.78–4.47) and an R^2^ of 0.15 (95% CI, −1.71 to 0.39). Although permutation testing suggested performance slightly better than chance (P_MAE = 0.044; P_R^2^ = 0.040), the R^2^ confidence interval overlapped zero, indicating that bacterial community composition alone provided relatively weak temporal resolution (Figure 5A).

The GC-MS metabolomics-based model achieved substantially better performance, with a held-out test MAE of 1.66 months (95% CI, 0.91–2.57) and an R^2^ of 0.72 (95% CI, 0.11–0.86). Both MAE and R^2^ were better than the permutation-based null distribution (both *p* < 0.001). Predicted values from this model were more closely aligned with the observed weathering times, supporting the stronger temporal information contained in metabolite profiles (Figure 5B).

The decision-level multi-omics model showed the lowest prediction error in internal cross-validation, with a held-out test MAE of 1.28 months (95% CI, 0.77–2.00) and an R^2^ of 0.82 (95% CI, 0.29–0.92) (Figure 5C). Its performance was better than the permutation-based null distribution (both *p* < 0.001). Compared with either single-omics model, the integrated model produced predictions that were more closely distributed around the 1:1 reference line. Residual analysis further showed that the bacterial community model had a broader error distribution, whereas the metabolomics and multi-omics models had more concentrated residuals (Figure S4). Feature-importance analysis of the random forest base models identified candidate bacterial and metabolic predictors contributing to weathering-time estimation (Figure S5). Within this dataset, metabolomic profiles outperformed bacterial community profiles, and decision-level integration further reduced prediction error.

## 4. Discussion

This study showed that empty puparia of *A. grahami* underwent measurable bacterial community and metabolomic changes during 13 months of indoor weathering. Metabolomic profiles showed clearer time-associated patterns than bacterial communities, particularly the consistent decrease in lipid-related metabolites. Conventional PMI_min_ estimation is mainly based on larval development [24], but such evidence is often unavailable in late-stage decomposition or skeletal remains [25]. Therefore, weathering-related molecular changes in empty puparia may provide supplementary information for estimating the PEI and supporting long-interval PMI_min_ assessment.

Previous studies on weathering time estimation of empty puparia have mainly focused on CHCs, which exhibit high chemical stability and show consistent changes in composition and relative abundance during weathering [26,27]. Analytical methods for CHCs are also well established, making them widely used indicators for estimating weathering time in forensic entomological evidence. However, the present study suggests that changes during the weathering of empty puparia are not limited to hydrocarbons but also involve multiple classes of lipid compounds. These findings indicate broader changes in lipid composition during the weathering process [28]. Among the 11 candidate time-dependent metabolic markers, lipids and lipid-like molecules were predominant, particularly long-chain fatty acids such as palmitic acid and stearic acid, which decreased consistently with increasing weathering time. This finding is consistent with previous reports on lipid degradation during biological decomposition and supports their potential as indicators of weathering time [29].

Further analysis showed that individual metabolites exhibited different trends over time during the weathering of empty puparia. Long-chain fatty acids, including stearic acid, palmitic acid, arachidic acid, and behenic acid, were abundant at the early stage (Mon1–3), decreased during the mid stage, and remained at low levels in the late stage (Mon7–13) [26,29,30]. In contrast, some metabolites, including linoleamide, tetracosanoic acid, and 1 monopalmitin, showed a different trend during the weathering of empty puparia. These compounds did not reach their highest levels at the earliest stage but instead increased during the mid stage (Mon3–5) and then gradually decreased over time. This observation suggests that weathering is not a simple linear degradation process but may involve the transient accumulation of intermediate products during lipid degradation. Previous studies have shown that complex lipids such as glycerides can be gradually converted into free fatty acids or amide derivatives during degradation. These intermediate products may accumulate temporarily at specific stages before further transformation by oxidative or other environmental processes. However, this remains a possible explanation and was not directly demonstrated in the present study [31,32,33].

In contrast to the trends described above, certain metabolites, such as cholesterol, exhibited high discriminative power in the PLS-DA model but showed relatively weak correlations with weathering time, failing to demonstrate stable time-dependent changes. This finding is consistent with previous observations on sterol compounds, which are relatively stable during decomposition but are susceptible to influences from initial biological variation and environmental factors [34,35]. Therefore, cholesterol is more likely to reflect overall differences among samples rather than serve as a reliable time-indicative marker. These results further suggest that reliance solely on multivariate statistical models for candidate-marker selection may introduce bias, and that correlation-based approaches should be incorporated for comprehensive evaluation. In addition, the metabolites significantly associated with weathering time in this study were predominantly lipids and lipid-like molecules, such as long-chain fatty acids and their derivatives, whereas positively correlated metabolites were relatively limited and mainly consisted of a small number of aromatic compounds. This finding differs from observations in some decomposition studies, where metabolites tend to accumulate during the later stages. A possible explanation is that empty puparia, as structurally resilient remains with low water content and limited nutrient availability, provide restricted substrates for further transformation, thereby limiting the sustained production and accumulation of secondary metabolites [30].

From a forensic application perspective, these findings indicate that long-chain fatty acids, such as stearic acid and palmitic acid, and their related derivatives show consistent decreases during the weathering of empty puparia, highlighting their potential as indicators of weathering time. In contrast, metabolites that increase during the mid stage, such as linoleamide and monoacylglycerols, may be useful for distinguishing intermediate stages of weathering. Therefore, integrating information from different classes of metabolites to construct multi-indicator models may improve the accuracy and robustness of weathering-time estimation, rather than relying on single metabolite markers.

At the bacterial level, this study observed temporal changes in bacterial communities associated with empty puparia during weathering. However, the overall magnitude of change was relatively limited. In general, the community structure did not exhibit pronounced or abrupt turnover, but rather gradual shifts in the relative proportions of dominant taxa, suggesting that the influence of weathering time on bacterial community structure was comparatively moderate. Moreover, after FDR correction, only one genus remained significantly associated with weathering time, indicating that genus-level bacterial indicators of temporal progression were limited under the conditions of this study. Therefore, relying solely on the bacterial community structure of empty puparia for long-term weathering-time estimation may be inherently limited.

Among the bacterial genera associated with weathering time, Bacillaceae-affiliated taxa and Staphylococcus increased in relative abundance during later weathering stages. Some members of these groups have been reported to utilize hydrophobic substrates or produce lipases [36,37,38,39]. In the present study, lipid-related metabolites, including fatty acids, long-chain alcohols, and esters, were also the main time-associated compounds. This pattern may indicate a potential ecological link between bacterial community shifts and the changing chemical composition of empty puparia. However, because the bacterial analysis was based on relative 16S rRNA gene abundance, this interpretation should be regarded as a possible explanation rather than direct evidence of absolute bacterial growth or bacterial lipid degradation.

The bacterial community–metabolite association should be interpreted cautiously because the significant Procrustes result was obtained only after selecting time-associated features from both datasets. Together with the non-significant partial Mantel test after controlling for weathering time, this suggests parallel responses to weathering time and environmental exposure rather than direct bacterial community–metabolite coupling. The limited concordance between bacterial and metabolomic changes suggests that these two datasets reflect different aspects of the weathering process.

Overall, the findings of this study indicate that changes in metabolite profiles during the weathering of empty puparia are primarily associated with transformations of intrinsic chemical components under environmental exposure, whereas bacterial community data may reflect compositional changes during exposure. These results suggest that, when empty puparia are used to estimate the PEI and support long-term PMI_min_ assessment, bacterial community and metabolome data reflect different aspects of the weathering process. Although they provide complementary information, their overall changes are not necessarily consistent.

These findings highlight a potential application of empty puparia as a source of temporal information in long-interval forensic investigations. When larval or pupal developmental evidence is no longer available, molecular changes preserved in empty puparia may help estimate the PEI and thereby add information to the entomological timeline. When combined with species identification, species-specific developmental data, relevant temperature records, and scene-specific information, the estimated PEI may provide supplementary evidence for PMI_min_ assessment. Although the multi-omics model yielded an MAE of 1.28 months, this value was obtained from sampling points separated by two months and should not be interpreted as one-month temporal precision in forensic casework. Rather, it indicates broad discrimination of post-eclosion weathering stages within this dataset, rather than precise timing in long-interval cases.

It should be noted that this study was conducted under a single uncontrolled indoor exposure condition using one emergence cohort; thus, the three pooled samples at each time point reflected within-cohort sampling variation rather than independent cohort or environmental replication. Indoor microenvironmental factors were not directly monitored, and extraction blanks, PCR negative controls, and environmental/container controls were not included, which is a major limitation for low-biomass empty puparial samples. Accordingly, the bacterial community profiles and prediction models should be interpreted cautiously. The reported MAE and R^2^ represent exploratory within-experiment performance rather than generalizable forensic performance. Although the multi-omics MAE of 1.28 months was lower than the two-month sampling interval, it should indicate broad stage discrimination within this dataset rather than one-month precision in long-interval casework. Future studies using independently reared cohorts, appropriate negative/environmental controls, and different exposure conditions are needed to assess the reproducibility and forensic applicability of these findings.

## 5. Conclusions

Empty puparia of *A. grahami* retain measurable molecular signatures during prolonged indoor weathering. The persistence of lipid-related temporal patterns suggests that chemical weathering of puparial remains may provide information on the PEI after adult emergence. These signatures may help bridge the evidential gap between completed insect development and evidence collection. When combined with species identification and developmental data, this information may supplement PMI_min_ assessment in long-interval cases where conventional larval evidence is no longer available. Future studies incorporating broader environmental settings, longer monitoring periods, and case-derived samples will further evaluate the robustness and practical forensic value of this approach.

## Data Availability

The 16S rRNA gene sequencing project has been registered in the NCBI BioProject database under accession number PRJNA1464520. Other data supporting the findings of this study are contained in the manuscript and Supplementary Materials or are available from the corresponding author upon reasonable request.

## Supplementary Materials

The following supporting information can be downloaded at: https://www.mdpi.com/article/10.3390/insects17070736/s1, Figure S1: Temporal variation in ambient temperature and relative humidity in Changsha from April 2024 to May 2025; Figure S2: Permutation validation of the PLS-DA model using 200 permutations; Figure S3: Dynamic changes of candidate time-associated metabolites during puparial weathering; Figure S4: Residual distribution of weathering-time prediction models; Figure S5: Candidate predictive features identified from the random forest base models; Figure S6: Indoor weathering setup and analytical workflow; Table S1: Correlation statistics, QC RSD values, and annotation parameters for the 13 candidate metabolites; Table S2: Raw peak intensity trends of the 13 candidate metabolites across weathering time; Table S3: Spearman correlations between candidate metabolites and weathering time after FDR correction. Table S4: Spearman correlations between candidate bacterial genera and weathering time after FDR correction.

## References

[1] Lutz L., Zehner R., Verhoff M.A., Bratzke H., Amendt J. (2021). It is all about the insects: A retrospective on 20 years of forensic entomology highlights the importance of insects in legal investigations. Int. J. Leg. Med..

[2] Matuszewski S., Mądra-Bielewicz A. (2016). Validation of temperature methods for the estimation of pre-appearance interval in carrion insects. Forensic Sci. Med. Pathol..

[3] Vanin S., Tuccia F., Pradelli J., Carta G., Giordani G. (2024). Identification of Diptera Puparia in Forensic and Archeo-Funerary Contexts. Insects.

[4] Thümmel L., Amendt J. (2026). Estimation of long term post mortem intervals by ATR-FTIR spectroscopy of weathered blow fly puparia. Spectrochim. Acta A Mol. Biomol. Spectrosc..

[5] Hood R.C. (2001). The effect of soil temperature and moisture on organic matter decomposition and plant growth. Isot. Environ. Health Stud..

[6] Metcalf J.L., Xu Z.Z., Weiss S., Lax S., Van Treuren W., Hyde E.R., Song S.J., Amir A., Larsen P., Sangwan N. (2016). Microbial community assembly and metabolic function during mammalian corpse decomposition. Science.

[7] Ochieng V.O., Osir E.O., Ochanda J.O., Olembo N.K. (1993). Temporal synthesis of cuticle proteins during larval development in *Glossina morsitans*. Comp. Biochem. Physiol. B.

[8] Zhu G.H., Yu X.J., Xie L.X., Luo H., Wang D., Lv J.Y., Xu X.H. (2013). Time of death revealed by hydrocarbons of empty puparia of *Chrysomya megacephala* (Fabricius) (Diptera: Calliphoridae): A field experiment. PLoS ONE.

[9] Zhu G.H., Xu X.H., Yu X.J., Zhang Y., Wang J.F. (2007). Puparial case hydrocarbons of *Chrysomya megacephala* as an indicator of the postmortem interval. Forensic Sci. Int..

[10] Zhang X., Bai Y., Ngando F.J., Qu H., Shang Y., Ren L., Guo Y. (2022). Predicting the Weathering Time by the Empty Puparium of *Sarcophaga peregrina* (Diptera: Sarcophagidae) with the ANN Models. Insects.

[11] Bajerlein D., Taberski D., Matuszewski S. (2018). Estimation of postmortem interval (PMI) based on empty puparia of *Phormia regina* (Meigen) (Diptera: Calliphoridae) and third larval stage of *Necrodes littoralis* (L.) (Coleoptera: Silphidae)—Advantages of using different PMI indicators. J. Forensic Leg. Med..

[12] Wydra J., Matuszewski S. (2021). The optimal post-eclosion interval while estimating the post-mortem interval based on an empty puparium. Forensic Sci. Med. Pathol..

[13] Zhu G.H., Jia Z.J., Yu X.J., Wu K.S., Chen L.S., Lv J.Y., Eric Benbow M. (2017). Predictable weathering of puparial hydrocarbons of necrophagous flies for determining the postmortem interval: A field experiment using *Chrysomya rufifacies*. Int. J. Leg. Med..

[14] Paula M.C., Michelutti K.B., Eulalio A., Piva R.C., Cardoso C.A.L., Antonialli-Junior W.F. (2018). New method for estimating the post-mortem interval using the chemical composition of different generations of empty puparia: Indoor cases. PLoS ONE.

[15] Qu H., Zhang X., Ye C., Ngando F.J., Shang Y., Yang F., Xiao J., Chen S., Guo Y. (2024). Combining spectrum and machine learning algorithms to predict the weathering time of empty puparia of *Sarcophaga peregrine* (Diptera: Sarcophagidae). Forensic Sci. Int..

[16] Pechal J.L., Crippen T.L., Tarone A.M., Lewis A.J., Tomberlin J.K., Benbow M.E. (2013). Microbial community functional change during vertebrate carrion decomposition. PLoS ONE.

[17] Xu J., Chen Q., Lønborg C., Li Y., Cai R., He C., Shi Q., Hu Y., Wang Y., Jiao N. (2022). You Exude What You Eat: How Carbon-, Nitrogen-, and Sulfur-Rich Organic Substrates Shape Microbial Community Composition and the Dissolved Organic Matter Pool. Appl. Environ. Microbiol..

[18] Liu Z., Han H., Meng F., Jiang Y., Cai J. (2020). Dynamic transcriptome profiling exploring cold tolerance in forensically important blow fly, *Aldrichina grahami* (Diptera: Calliphoridae). BMC Genom..

[19] Chen W., Yang L., Ren L., Shang Y., Wang S., Guo Y. (2019). Impact of Constant Versus Fluctuating Temperatures on the Development and Life History Parameters of *Aldrichina grahami* (Diptera: Calliphoridae). Insects.

[20] Yadong G., Jifeng C., Zhenchu T., Xiong F., Zhang L., Fu Y., Jianbo L., Yaoqing C., Fanming M., Jifang W. (2011). Application of *Aldrichina grahami* (Diptera, Calliphoridae) for forensic investigation in central-south China. Rom. J. Leg. Med..

[21] Hönigschnabl S., Schaden E., Stichenwirth M., Schneider B., Klupp N., Kremeier E., Lehner W., Vycudilik W., Bauer G., Risser D. (2002). Discovery of decomposed and mummified corpses in the domestic setting—A marker of social isolation?. J. Forensic Sci..

[22] Ceciliason A.S., Andersson M.G., Lindström A., Sandler H. (2018). Quantifying human decomposition in an indoor setting and implications for postmortem interval estimation. Forensic Sci. Int..

[23] Chen L. (2013). Corpse-Feeding Flies in China.

[24] Amendt J., Campobasso C.P., Gaudry E., Reiter C., LeBlanc H.N., Hall M.J. (2007). Best practice in forensic entomology—Standards and guidelines. Int. J. Leg. Med..

[25] Bansode S., Morajkar A., Ragade V., More V., Kharat K. (2025). Challenges and considerations in forensic entomology: A comprehensive review. J. Forensic Leg. Med..

[26] Stewart-Yates D., Maker G.L., D′Errico S., Magni P.A. (2025). Advances and Current Status in the Use of Cuticular Hydrocarbons for Forensic Entomology Applications. Insects.

[27] Moore H.E., Pechal J.L., Benbow M.E., Drijfhout F.P. (2017). The potential use of cuticular hydrocarbons and multivariate analysis to age empty puparial cases of *Calliphora vicina* and *Lucilia sericata*. Sci. Rep..

[28] Howard R.W., Blomquist G.J. (2005). Ecological, behavioral, and biochemical aspects of insect hydrocarbons. Annu. Rev. Entomol..

[29] Frere B., Suchaud F., Bernier G., Cottin F., Vincent B., Dourel L., Lelong A., Arpino P. (2014). GC-MS analysis of cuticular lipids in recent and older scavenger insect puparia. An approach to estimate the postmortem interval (PMI). Anal. Bioanal. Chem..

[30] Moore H., Lutz L., Bernhardt V., Drijfhout F.P., Cody R.B., Amendt J. (2022). Cuticular hydrocarbons for the identification and geographic assignment of empty puparia of forensically important flies. Int. J. Leg. Med..

[31] Frick A.A., Weyermann C. (2019). An untargeted lipidomic approach for qualitative determination of latent fingermark glycerides using UPLC-IMS-QToF-MS(E). Analyst.

[32] Jamil M.A.R., Siddiki S., Touchy A.S., Rashed M.N., Poly S.S., Jing Y., Ting K.W., Toyao T., Maeno Z., Shimizu K.I. (2019). Selective Transformations of Triglycerides into Fatty Amines, Amides, and Nitriles by using Heterogeneous Catalysis. ChemSusChem.

[33] Kimura W., Endo Y. (2017). Decomposition products of glycidyl esters of fatty acids by heating. Biosci. Biotechnol. Biochem..

[34] Busch T.P., King A.J. (2010). Stability of Cholesterol, 7-Ketocholesterol and β-Sitosterol during Saponification: Ramifications for Artifact Monitoring of Sterol Oxide Products. J. Am. Oil Chem. Soc..

[35] Giorgi V., Menéndez P., García-Carnelli C. (2019). Microbial transformation of cholesterol: Reactions and practical aspects—An update. World J. Microbiol. Biotechnol..

[36] Parthipan P., Preetham E., Machuca L.L., Rahman P.K., Murugan K., Rajasekar A. (2017). Biosurfactant and Degradative Enzymes Mediated Crude Oil Degradation by Bacterium Bacillus subtilis A1. Front. Microbiol..

[37] Sharma S., Pandey L.M. (2020). Production of biosurfactant by Bacillus subtilis RSL-2 isolated from sludge and biosurfactant mediated degradation of oil. Bioresour. Technol..

[38] Xie W., Khosasih V., Suwanto A., Kim H.K. (2012). Characterization of lipases from Staphylococcus aureus and Staphylococcus epidermidis isolated from human facial sebaceous skin. J. Microbiol. Biotechnol..

[39] Chen X., Alonzo F. (2019). Bacterial lipolysis of immune-activating ligands promotes evasion of innate defenses. Proc. Natl. Acad. Sci. USA.

