# Supplementary material for: Metabolomic and Bacterial Community Signatures of Weathering Time in Empty Puparia of Aldrichina grahami (Aldrich, 1930) (Diptera: Calliphoridae)
Source: Insects. 2026 Jul 17;17(7):736. doi: 10.3390/insects17070736 (PMC13411882; doi:10.3390/insects17070736)
Supplement: Supplementary file 1 [file insects-17-00736-s001.zip › Supplementary_Tables.pdf]

Supplementary Table S1. Correlation statistics, QC RSD values, and annotation parameters for the 13 candidate metabolites.

Metabolite	RT (min)	RI	Library RI	|Delta RI|	Match score	RIScore	QC RSD (%)	Spearman r	P value	q value	Annotation level
Stearic Acid	9.875	2231.75	2243.00	11.25	88.7	99.65	25.76	-0.900	<0.001	<0.001	MSI level 2
Palmitic Acid	8.970	2036.25	2049.00	12.75	89.4	99.57	15.46	-0.747	<0.001	0.001	MSI level 2
Cholesterol	14.212	3165.69	3165.00	0.69	96.7	99.98	10.82	-0.609	0.004	0.014	MSI level 2
Petroselinic Acid	9.766	2207.03	2211.00	3.97	92.6	99.88	33.89	-0.932	<0.001	<0.001	MSI level 2
Arachidic Acid	10.721	2429.51	2443.00	13.49	89.6	99.62	18.35	-0.735	<0.001	<0.001	MSI level 2
Behenic Acid	11.503	2625.26	2636.00	10.74	88.6	99.72	17.24	-0.751	<0.001	<0.001	MSI level 2
1-Hexacosanol	12.811	2927.84	2940.00	12.16	86.6	99.71	18.40	-0.818	<0.001	<0.001	MSI level 2
Tetracosanoic Acid	12.312	2821.71	2817.00	4.71	88.7	99.88	20.17	-0.743	<0.001	0.002	MSI level 2
1-Monopalmitin	11.273	2566.75	2606.00	39.25	95.9	98.96	15.29	-0.723	<0.001	0.002	MSI level 2
Carbonic Acid, Eicosyl Vinyl Ester	10.983	2493.41	2497.00	3.59	96.6	99.90	9.50	-0.790	<0.001	<0.001	MSI level 2
Glycerol Monostearate	12.038	2758.03	2768.00	9.97	95.4	99.75	16.52	-0.759	<0.001	0.001	MSI level 2
L-Arabinitol	7.489	1750.27	1739.00	11.27	91.5	99.55	4.73	-0.260	0.251	0.361	MSI level 2
Linoleamide	10.629	2407.07	2398.00	9.07	91.6	99.74	18.72	-0.857	<0.001	<0.001	MSI level 2

Note: RT, retention time; RI, experimental retention index; |Delta RI|, absolute difference between experimental RI and library RI. QC RSD values are shown as percentages. Metabolites were putatively annotated based on spectral library and retention-index matching and are reported as MSI level 2 annotations. The direction of change was assigned according to the overall abundance trend with increasing weathering time.

Supplementary Table S2. Raw peak intensity trends of the 13 candidate metabolites across weathering time.

Metabolite	Mon1	Mon3	Mon5	Mon7	Mon9	Mon11	Mon13	Overall trend
Stearic Acid	108.06 ± 6.18	89.27 ± 5.00	81.63 ± 1.92	81.39 ± 7.44	70.93 ± 9.69	63.60 ± 9.40	59.84 ± 4.49	Decreasing
Palmitic Acid	113.50 ± 6.99	106.89 ± 8.97	107.61 ± 2.42	96.26 ± 1.62	87.90 ± 12.90	82.25 ± 12.52	84.22 ± 3.33	Decreasing
Cholesterol	10.87 ± 2.39	9.72 ± 5.30	17.37 ± 11.60	1.46 ± 0.17	2.22 ± 2.04	1.54 ± 0.59	3.63 ± 1.31	Weak decreasing
Petroselinic Acid	16.20 ± 3.75	9.14 ± 2.92	5.10 ± 0.48	3.66 ± 0.23	3.74 ± 0.42	2.88 ± 0.49	2.83 ± 0.23	Decreasing
Arachidic Acid	16.24 ± 0.86	18.20 ± 1.45	16.28 ± 3.04	8.97 ± 1.32	11.03 ± 1.62	8.99 ± 0.57	8.95 ± 2.97	Decreasing
Behenic Acid	15.67 ± 1.59	17.24 ± 1.44	14.29 ± 1.28	8.95 ± 1.97	10.01 ± 0.81	8.30 ± 1.34	8.61 ± 3.17	Decreasing
1-Hexacosanol	11.83 ± 2.90	11.49 ± 0.37	8.60 ± 2.57	4.08 ± 0.59	6.62 ± 1.58	3.17 ± 0.77	3.97 ± 0.87	Decreasing
Tetracosanoic Acid	7.92 ± 0.77	9.72 ± 1.32	8.21 ± 1.77	6.18 ± 1.44	5.22 ± 0.34	4.17 ± 0.69	5.23 ± 1.62	Decreasing
1-Monopalmitin	24.77 ± 0.34	21.29 ± 1.19	20.89 ± 0.58	26.51 ± 1.40	22.12 ± 2.81	20.89 ± 2.11	19.99 ± 2.68	Decreasing
Carbonic Acid, Eicosyl Vinyl Ester	4.86 ± 0.25	4.74 ± 0.60	4.03 ± 0.28	2.38 ± 1.00	3.02 ± 0.82	2.97 ± 1.13	2.28 ± 0.49	Decreasing
Glycerol Monostearate	14.91 ± 0.04	12.80 ± 0.69	12.31 ± 0.48	15.45 ± 0.63	12.68 ± 1.75	12.06 ± 1.29	11.53 ± 1.56	Decreasing
L-Arabinitol	36.08 ± 1.89	36.85 ± 2.79	37.30 ± 2.18	44.03 ± 3.06	36.98 ± 2.99	36.88 ± 3.11	40.52 ± 4.55	No consistent trend
Linoleamide	2.10 ± 0.14	4.50 ± 1.50	2.83 ± 0.92	1.51 ± 0.13	1.62 ± 0.10	1.03 ± 0.55	0.87 ± 0.16	Decreasing

Note: Values are raw peak intensities shown as mean ± SD across three pooled samples at each time point and scaled by 10^6 for readability. Mon1, Mon3, Mon5, Mon7, Mon9, Mon11, and Mon13 indicate 1, 3, 5, 7, 9, 11, and 13 months after adult emergence, respectively.

Supplementary Table S3. Spearman correlations between candidate metabolites and weathering time after FDR correction.

Feature	Spearman r	P value	FDR-adjusted P (q)
Stearic Acid	-0.900	<0.001	<0.001
Palmitic Acid	-0.747	<0.001	0.001
Petroselinic Acid	-0.932	<0.001	<0.001
Octadecanamide	-0.869	<0.001	<0.001
Linoleamide	-0.857	<0.001	<0.001
Tetracosanol	-0.853	<0.001	<0.001
Glycerol Monostearate	-0.759	<0.001	0.001
Arachidic Acid	-0.735	<0.001	<0.001
Behenic Acid	-0.751	<0.001	<0.001
Tetracosanoic Acid	-0.743	<0.001	0.002
1-Monopalmitin	-0.723	<0.001	0.002

Note: P values were calculated using Spearman correlation analysis and adjusted using the Benjamini-Hochberg false discovery rate (FDR) method. The listed metabolites are candidate time-associated metabolites highlighted in the manuscript.

Supplementary Table S4. Spearman correlations between candidate bacterial genera and weathering time after FDR correction.

Feature	Spearman r	P value	FDR-adjusted P (q)
unclassified_f_Bacillaceae	0.668	0.002	0.091
Lederbergia	0.643	0.002	0.091
norank_f_Bacillaceae	0.584	0.008	0.127
Staphylococcus	0.590	0.006	0.116
unclassified_f_Micrococcaceae	-0.861	<0.001	0.016
Pseudochrobactrum	-0.610	0.003	0.091
Paenalcaligenes	-0.598	0.003	0.092
Paenochrobactrum	-0.582	0.005	0.116

Note: P values were calculated using Spearman correlation analysis and adjusted using the Benjamini-Hochberg false discovery rate (FDR) method. FDR-supported associations were defined as $q < 0.05$; nominal trends did not remain significant after FDR correction.
