# Supplementary material for: Metabolomic and Bacterial Community Signatures of Weathering Time in Empty Puparia of Aldrichina grahami (Aldrich, 1930) (Diptera: Calliphoridae)
Source: Insects. 2026 Jul 17;17(7):736. doi: 10.3390/insects17070736 (PMC13411882; doi:10.3390/insects17070736)
Supplement: Supplementary file 1 [file insects-17-00736-s001.zip › supplementary-figure.pdf]

Supplementary Information:

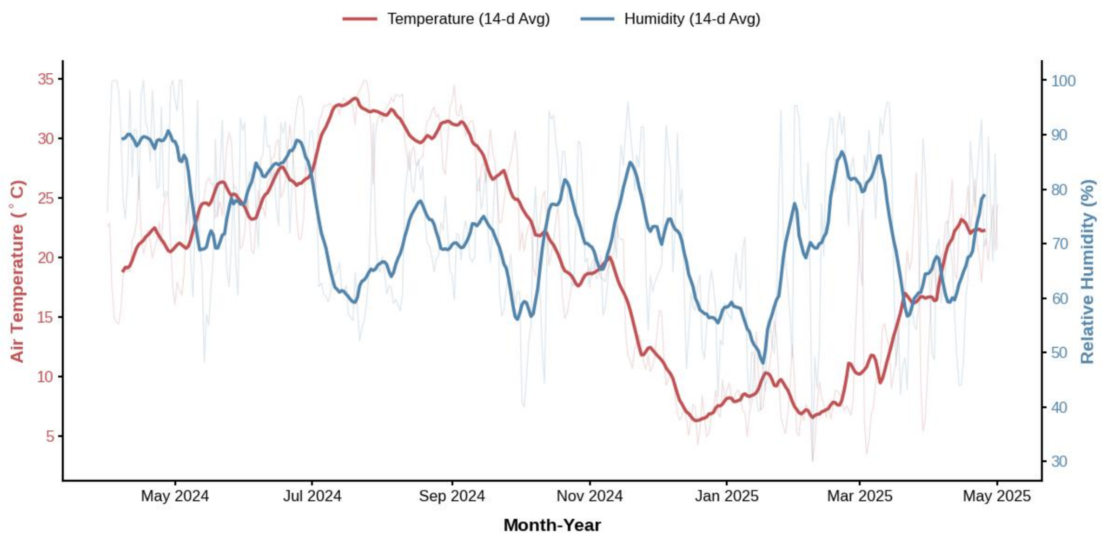

Figure S1. Temporal variation in ambient temperature and relative humidity in Changsha from April 2024 to May 2025. The thick solid lines represent 14-day moving average trends, while the semi-transparent thin lines indicate daily mean values. Meteorological data were obtained from the Changsha Xingsha Meteorological Station.

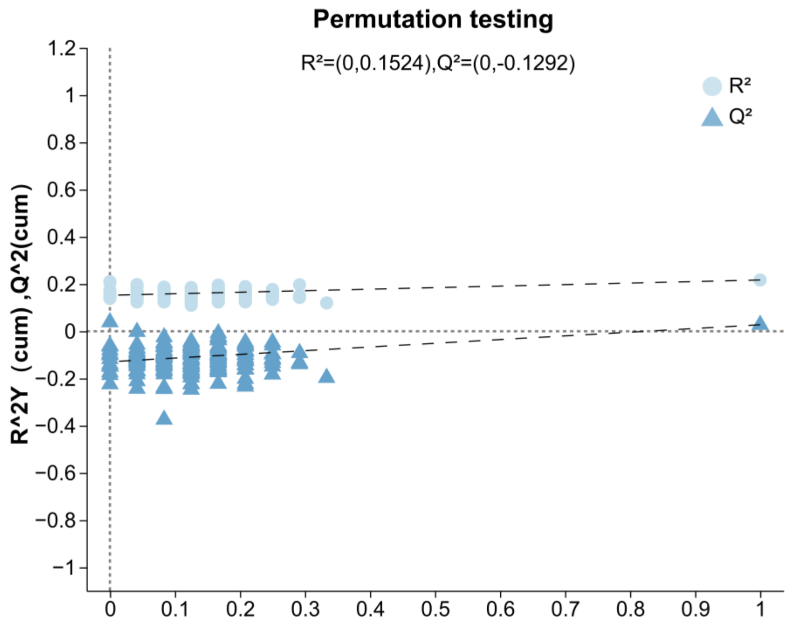

Figure S2. Permutation validation of the PLS-DA model using 200 permutations. The Q^2 intercept was negative (-0.1292), while the low Q^2 value indicated limited predictive ability.

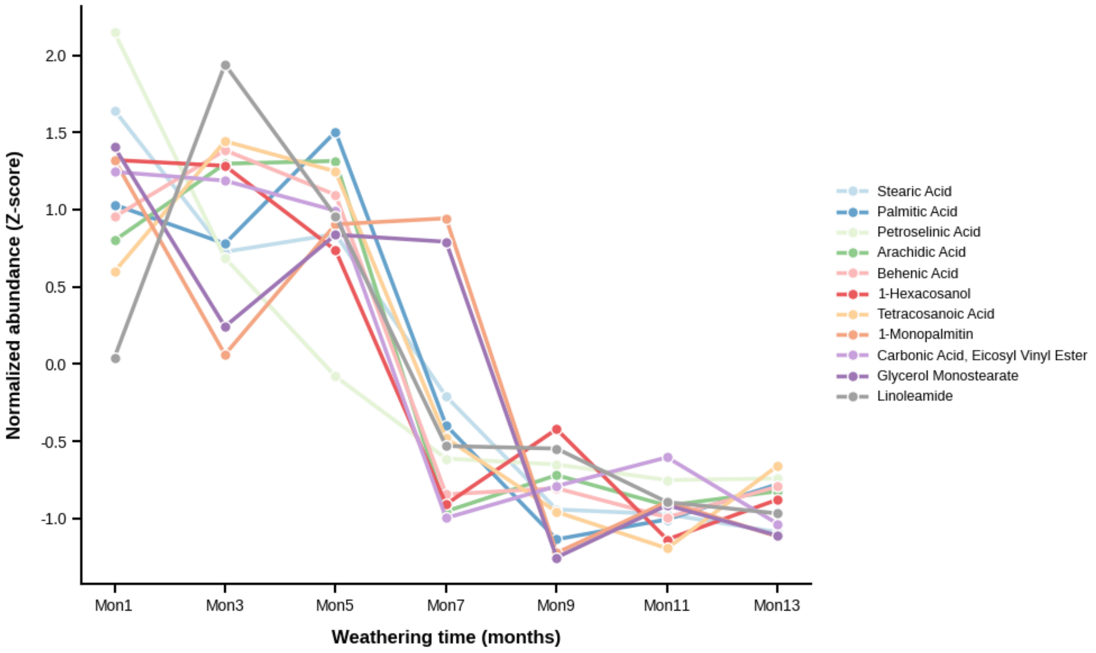

Figure S3. Dynamic changes of candidate time-associated metabolites during puparial weathering. Relative abundance profiles of 11 candidate time-associated metabolites jointly identified by PLS-DA and Spearman correlation analysis across different weathering intervals.

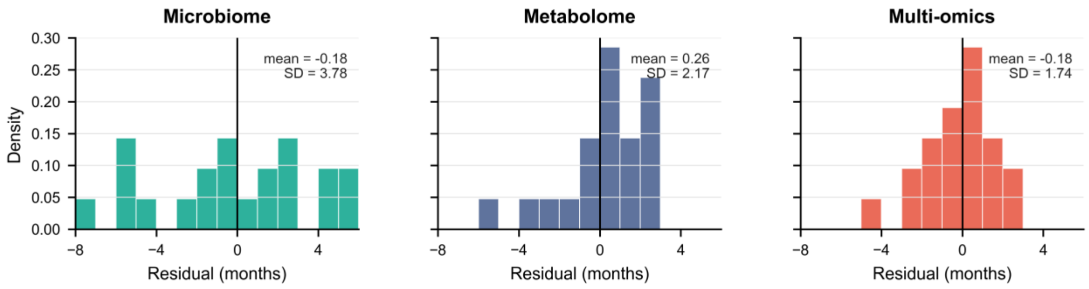

Figure S4. Residual distribution of weathering-time prediction models. Residual distributions of the bacterial community-based, metabolomics-based, and multi-omics models based on held-out test-set predictions. Residuals were calculated as predicted minus observed weathering time. The vertical black line indicates zero residual. A narrower distribution around zero indicates higher prediction stability.

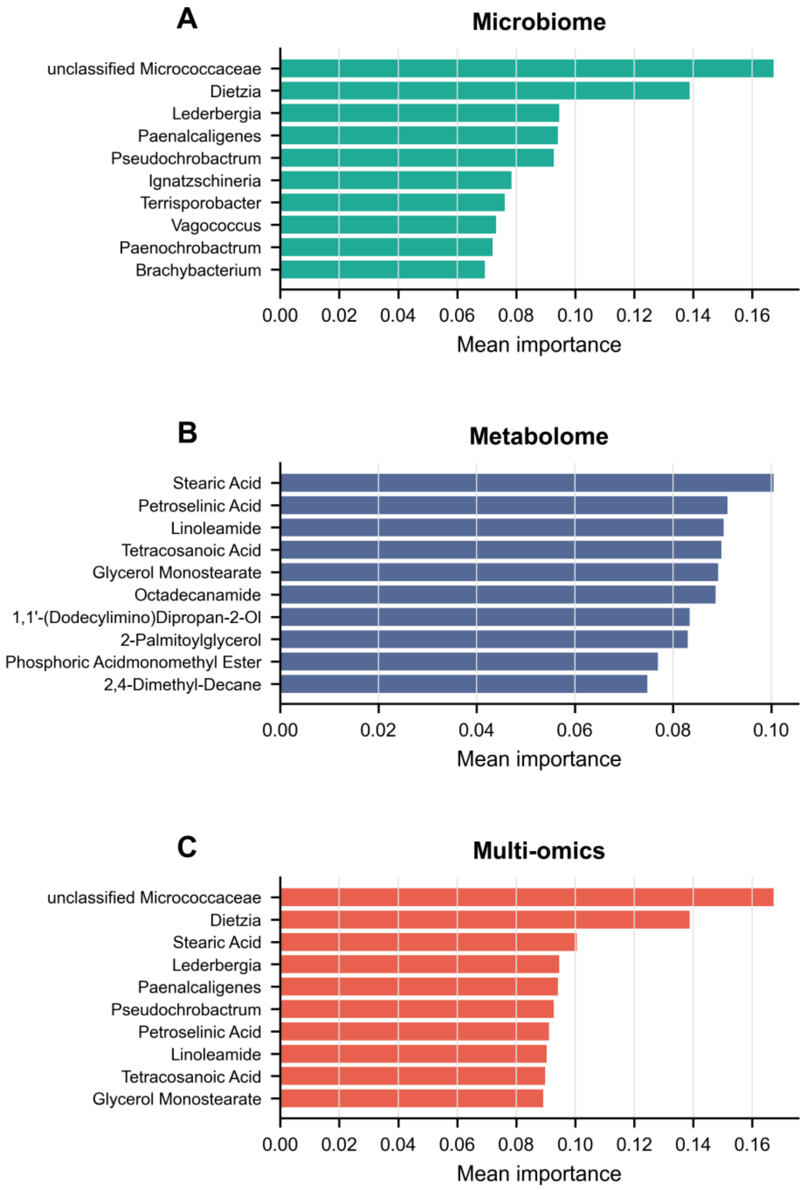

Figure S5. Candidate predictive features identified from the random forest base models. (A) Mean feature importance values from the bacterial community-based random forest model. (B) Mean feature importance values from the GC-MS metabolomics-based random forest model. (C) Microbial and metabolic features contributing to the decision-level multi-omics framework, summarized from the corresponding random forest base models across cross-validation folds. Feature importance values were averaged across folds.

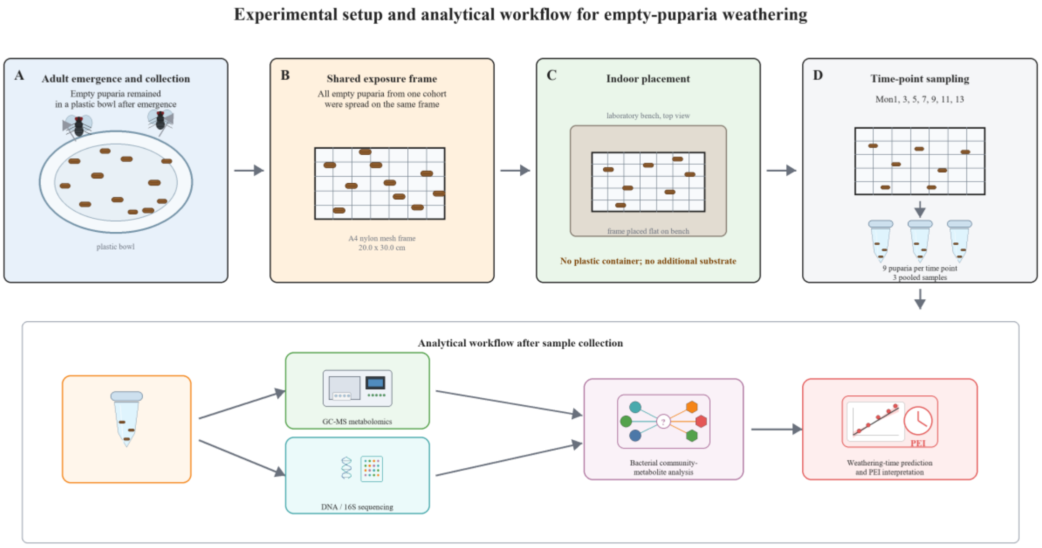

Figure S6. Indoor weathering setup and analytical workflow. (A) Empty puparia remained in a plastic bowl after adult emergence. (B) Empty puparia were spread on an A4-sized nylon mesh frame. (C) The mesh frame was placed flat on a laboratory bench without plastic containers or additional substrate. (D) At each time point, empty puparia were collected and pooled for GC-MS metabolomics and 16S rRNA gene sequencing.
